# Racial Discrimination is Associated with Binge-Eating Disorder in Early Adolescents: A Cross-Sectional Analysis

**DOI:** 10.21203/rs.3.rs-2973069/v1

**Published:** 2023-05-31

**Authors:** Julia H Raney, Abubakr A Al-shoaibi, Iris Y. Shao, Kyle T Ganson, Alexander Testa, Dylan B. Jackson, Jinbo He, David V. Glidden, Jason M. Nagata

**Affiliations:** University of California, San Francisco; University of California, San Francisco; University of California, San Francisco; University of Toronto; The University of Texas Health Science Center at Houston; Johns Hopkins University; Chinese University of Hong Kong, Shenzhen; University of California, San Francisco; University of California, San Francisco

**Keywords:** Racial discrimination, binge-eating disorder, adolescent health

## Abstract

**Background:**

Racial and ethnic discrimination are known stressors and are associated with negative psychological and physical health outcomes. Previous studies have found relationships between racial/ethnic discrimination and binge-eating disorder (BED), though they have mainly focused on adult populations. The aim of this study was to determine associations between racial/ethnic discrimination and BED in a large, national cohort study of early adolescents. We further sought to explore associations between the racial/ethnic discrimination perpetrator (students, teachers, or other adults) and BED.

**Methods:**

We analyzed cross-sectional data from the Adolescent Brain Cognitive Development Study (ABCD) (N = 11,075, 2018–2020). Logistic regression analyses examined associations between self-reported racial or ethnic discrimination and binge-eating behaviors and diagnosis. Racial/ethnic discrimination measures were assessed based on the Perceived Discrimination Scale, which measures experiences of discrimination based on race/ethnicity and frequency of ethnic discrimination by teachers, adults outside of school, and students. Binge-eating behaviors and diagnosis were based on the Kiddie Schedule for Affective Disorders and Schizophrenia (KSAD-5), adjusting for age, sex, race/ethnicity, household income, parental education, and site.

**Results:**

In this racially diverse sample of adolescents (N = 11,075, mean age: 11 years), 4.7% of adolescents reported racial or ethnic discrimination and 1.1% met the criteria for BED at the one-year follow-up. In the adjusted models, racial/ethnic discrimination was associated with 3 times higher odds of having BED (OR 3.31, CI 1.66–7.74); when investigating associations between the racial/ethnic discrimination perpetrator (students, teachers, or other adults) and BED, experiencing ethnic discrimination by students and adults outside school were associated with significantly increased odds of BED diagnosis (OR 1.36, CI 1.10–1.68 & OR 1.42 CI 1.06–1.90, respectively); further, increased odds of binge eating behaviors was only significantly associated with ethnic discrimination perpetuated by students (OR 1.12, CI 1.02–1.23).

**Conclusions:**

Children and adolescents who have experienced racial/ethnic discrimination, particularly when discrimination was perpetuated by other students, have higher odds of having binge-eating behaviors and diagnoses. Clinicians may consider screening for racial discrimination and providing anti-racist, trauma-informed care when evaluating and treating patients for BED.

## Plain English Summary

Binge-eating disorder is associated with significant psychological and physical consequences including depression, anxiety, impaired relationships, and obesity. Recent research has demonstrated that many of these behaviors develop in early adolescence, a time of immense psychosocial development. Racial and ethnic discrimination are known stressors, and previous studies have found relationships between racial and ethnic discrimination and binge-eating disorder, though they have mainly focused on adult populations. This study helps fill that gap by using data from the Adolescent Brain Cognitive Development Study (ABCD) Study, the largest prospective study of adolescent brain development in the US. In this large, racially diverse, national study of 11,075 adolescents aged primary 11 – 12 years old, we find that early adolescents who have experienced racial/ethnic discrimination, particularly when discrimination was perpetuated by other students, have higher odds of having binge-eating behaviors and diagnoses. These findings have important school and clinical implications. For example, schools may consider implementing curricula focused on anti-racist practices that foster environments where all youth to thrive. In addition, we recommend that clinicians screen for racial discrimination and provide culturally sensitive, equity-focused care when evaluating and treating patients with binge-eating disorder.

## Background

Binge-eating disorder (BED), characterized by consuming a large amount of food while feeling a loss of control and negative emotions,(1) is estimated to affect 4.5% of the population across the lifetime,(2) with those from minoritized backgrounds experience even higher rates of BED(3) and binge-eating behaviors.(4) BED is associated with significant psychological and physical consequences including depression, anxiety, impaired relationships, and obesity.(2, 5, 6) Given BED’s prevalence, consequences, and challenges with accessing treatment, identifying risk factors for the development of BED is critical to promote health and health equity.

Racial discrimination, or personally mediated racism, has been recognized as a core driver of health inequities in adolescents and children.(7–9) Personally mediated racism includes experiences of stereotypes and prejudices about a person’s ability, intent, or motives on the basis of race.(10) Personally mediated racism can be expressed implicitly or explicitly and can be experienced directly or indirectly. Minoritized children and adolescents face personally mediated racism in their interactions with teachers and students at school, during extracurricular activities, and increasingly in online, digital environments. (11, 12) Experiencing racial harassment and taunts can over-activate the stress response, and have cascading effects including increased and prolonged levels of exposure to stress hormones and oxidative stress.(13) A growing body of literature has found associations between racial discrimination and BED. Several studies of Latino and African American adults have demonstrated significant associations between racial discrimination and binge eating.(14–16) For example, a US nationally representative sample of Latino individuals found a significant association with discrimination and binge eating, with the average age in the sample being 40 years old (range 18–97).(15) In addition, Assari et al.’s paper demonstrated significant associations between perceived discrimination and BED in a nationally representative example of African American adults (average age 42).(16) Studies have also significant associations between maladaptive eating behaviors in young adult Black women (ages 18–25)(17) and Latino young adults (ages 18–25).(18)

However, little is known about the association between racial discrimination and the development of BED in early adolescents. A better understanding of the development of BED in this age-group is especially critical for several reasons. First, recent research has demonstrated that many of these behaviors develop in early adolescents, a time of immense psychosocial development.(19) A study of 10- to 11-year old children from the ABCD Study, a large, diverse, population-based sample, estimated the prevalence of BED to be 1.1%.(20) In addition, a population-based study of 14 year old early adolescents found a 14% prevalence rate of subclinical binge-eating behaviors.(21) Second, early adolescents experience racism and discrimination at unacceptable rates; a recent study estimated that 4.8% of 10- to 11-year old children reported being treated unfairly because of their race, ethnicity or color, and 10% of Black children reported experiencing racism.(22) Given the prevalence of perceived discrimination experienced by youth in this age group, it is critical to characterize the public health effects and rapidly implement antiracism practices. Given the significant impact that peers, teachers, and non-caregiver adults have on early adolescent development,(23–25) we further sought to characterize the impacts of expressed by these varying groups.

The purpose of the current study was to examine the associations between racial discrimination and BED among a large, diverse cohort of early-adolescents ages 9–14 years old (primarily ages 10–11).

## Methods

### Study population

This study uses survey data from the Adolescent Brain Cognitive Development (ABCD) Study to determine the association between racial discrimination and BED among US early adolescents. The ABCD Study is a large, prospective cohort study of brain development, health, and health behaviors among US adolescents across 21 recruitment sites.(26) Study design and recruitment strategies have previously been described.(27) We included data collected between 2018–2020, corresponding to Year one of the ABCD Study, the first year adolescent-reported racial discrimination was assessed. Participants missing data for i) sociodemographic characteristics or ii) all discrimination questions (n = 710) were excluded, yielding the total sample of 11,075. All participants gave assent, and parents/caregivers provided signed informed consent. The ABCD Study protocol was approved by the Institutional Review Board of the University of California, San Diego and at each respective study site.

### Exposure: Racial discrimination

Racial discrimination was measured using the Perceived Discrimination Scale,(28,29) which was developed to measure adolescents’ perception of being unaccepted in society or being unwanted based on their racial or ethnic background or skin color. Adolescents were asked, “In the past 12 months, have you felt discriminated against: because of your race, ethnicity, or color?” In addition, adolescents were asked how often they had been treated unfairly or negatively because of their ethnic background by each of the following groups: teachers, adults outside the school, and students (1 = almost never; 2 = rarely; 3 = sometimes; 4 = often; 5 = very often).

### Outcome: Binge-eating disorder

BED diagnosis and behaviors were assessed that the one-year follow-up through parent/caregiver responses to the Kiddie Schedule for Affective Disorders and Schizophrenia (KSADS-5), a computerized tool developed to categorize child and adolescent mental health based on the DSM-5. (1,30) Parents/caregivers completed all modules of the KSADS-5 to characteristics, frequency, and duration of their child’s binge-eating behaviors as well as associated distress. The presence of binge-eating behaviors was assessed by asking if their child experienced a loss of control of their eating and ate way more than he/she needed. BED was determined using the KSADS-5 computerized scoring system, where responses to the survey questions were extrapolated into their respective diagnosis based on reported behaviors corresponding to the DSM-5. Although bulimia nervosa (BN) also consists of binge eating symptoms, the prevalence of BN in the sample was low and therefore this study focused on binge-eating behaviors and BED.

### Covariates

We selected potential confounders for the association between racial discrimination and BED based on prior literature and theory.(16,31) Age, sex (male, female), race/ethnicity (White, Latino/Hispanic, Black, Asian, Native American, Other), nativity (youth born in US or outside of the US), household income ($24,999 or less, $25,000 through $49,999, $50,000 through $74,999, $75,000 through $99,999, $100,000 through $199,999, $200,000 and greater), and highest parent education (high school or less vs. college or more) were selected from parent self-report data at baseline. ABCD Study site (21 total sites) was also included to adjust for potential regional variation.

### Statistical analyses

Unadjusted and adjusted logistic regressions were conducted using Stata 17.0 (StataCorp, College Station, TX) to estimate associations between past year experiences of racial/ethnic discrimination and BED diagnosis and behaviors. In addition, unadjusted and adjusted logistic regression analyses estimated the association between frequency of ethnic discrimination (teachers, adults outside school, students), and BED diagnosis and behaviors. The ABCD study sociodemographic varaiabeles were standardized to match the distribution American Community Survey from the U.S. Census.(32)

## Results

The 11,075 adolescent respondents were racially and ethnically diverse (53.4% White, 19.6% Latino/Hispanic, 16.5% Black, 5.6% Asian, 3.2% Native American, 1.4% Other, Table 1). Binge-eating behaviors and diagnosis were relative rare at 7.9% and 1.1%, respectively. Approximately one in twenty youth reported experiencing racial or ethnic discrimination in the past year. In addition, adolescents reported higher rates of students perpetuating ethnic discrimination that teachers or other adults outside of school.

In both the adjusted and unadjusted models, racial/ethnic discrimination was associated with increased binge-eating behaviors and binge-eating disorder diagnosis (Table 2). In the adjusted models, adolescents who reported perceived discrimination had 3.31 higher odds of BED (95% CI 1.66 – 6.63). Increased frequency of ethnic discrimination by students was also significantly associated with a higher odds of BED diagnosis and behaviors. In addition, respondents who reported more frequent ethnic discrimination by adults outside of school had significantly higher odds of BED diagnosis.

## Discussion

In this national, sociodemographically diverse sample of early adolescents in the U.S., we found that experiencing racial/ethnic discrimination was associated with binge-eating behaviors and diagnosis, even when adjusting for confounding factors including race, sex, nativity, parental education, and socioeconomic status.

The relationship between discrimination and binge-eating is consistent with prior studies in minoritized adult populations that have demonstrated associations between experiencing racial/ethnic discrimination and binge-eating behaviors in Latino and Black the general adult population and young adults.(14, 16) Our findings contribute to the literature by demonstrating that perceived discrimination is significantly associated with higher odds of binge-eating behaviors and diagnosis in a national, diverse population of US early adolescents; importantly, early-adolescents represent an under-researched age group whose developmental period is vulnerable to developing health-related risk behaviors.(19, 33) As BED is associated with significant distress, morbidity, and mortality, it is critical to investigate risk factors in this age group to design primary and secondary prevention interventions.(34, 35)

A potential mechanism of this relationship is experiencing discrimination may impact adolescents’ self-esteem and increases risk for depression symptoms, both of which are associated with increased rates of BED.(28, 36, 37) In addition, several theoretical models have conceptualized racial/ethnic discrimination as an important stressor that drives binge eating from maladaptive coping responses from the resultant increased stress and changes in cortisol levels.(38, 39)

Our study further adds to the literature by exploring how unique groups of perpetrators influence the association between discrimination and BED. In our study, adolescents reported students to be the most common perpetuators of ethnic discrimination with one if four adolescents reporting experiencing ethnic discrimination by students rarely or more frequently; in addition, reporting ethnic discrimination perpetuated by students was significantly associated with increased odds of binge-eating disorder behaviors and diagnosis. The significant impact of peer discrimination on adolescent’s mental health has been supported in prior literature.(24, 40) From a developmental perspective, peer discrimination may be particularly impactful for early adolescents as they increasing spend time outside of the home and rely more on peers for psychosocial acceptance, self-concept, socialization, and identity formation.(41, 42) Several studies have shown that peer victimization in early adolescence is predictive of subsequent development of depressive symptoms.(43, 44) Our study builds upon these studies by highlighting that discrimination is also associated with BED in a national, diverse sample of early adolescents. Of note, experiencing discrimination by other adults outside school was also associated with a significantly higher odds of BED diagnosis. This is consistent with literature that shows the important influence that nonparental adults, such as mentors and police, can have on adolescent mental health.(45–47) Importantly, in the adjusted models, discrimination perpetuated by teachers was not significantly associated with binge-eating behaviors or diagnosis and discrimination by adults outside school was not associated with binge eating symptoms. However, this may be partially due to the smaller sample size in these groups. Prior studies have found that teachers play a critical role in adolescent development and mental health.(48, 49) Further studies should continue to investigate the relationships between ethnic discrimination perpetuated by teachers and adults outside school and binge eating.

This study has several limitations. This study used parent report of binge eating diagnosis and behaviors. While parents are important reporters for eating disorders in early adolescents as children have less insight into their eating behaviors,(26, 50) parent and child reports of binge-eating behaviors have a tendency of low concordance.(26, 50, 51) In addition, this study did not explore the setting where the discrimination occurred so we are unable to assess how the location (ie school, virtual settings, community) estimate the impacts BED behaviors.

## Conclusions

This study demonstrates that experiencing racial or ethnic discrimination in early adolescence is associated with BED diagnosis and symptoms, which has important school, clinical, and public policy implications. For example, schools may consider implementing curricula focused on anti-racist practices that foster environments where all youth to thrive.(52) In addition, minoritized populations, for instance, have historically received inadequate access to eating disorder care and inclusion in eating disorders research, which increases the risk of delayed and poorer outcomes.(53) Policy changes that that target these systemic issues, such as increased education about BED among diverse populations, increased food assistance among marginalized communities,(54) and increased access to eating disorder trained mental health professionals,(53) may profoundly impact the risk of BED. The US Preventive Services Task Force (USPSTF) recently reviewed eating disorder screening in asymptomatic adolescents and adults and determined there to be insufficient evidence to recommend routine screening in this population, especially among racial/ethnic minority populations (55). However, clinicians may still consider screening for eating disorder behaviors in early adolescents with significant risk factors, such as racial discrimination, given the significant physician and mental health consequences of eating disorders.(56)

## Figures and Tables

**Table 1:** Sociodemographic characteristics of participants in the Adolescent Brain Cognitive Development Study (ABCD) Study, 2018–2020, (n=11,075)

	Mean (SD) or %
**Demographic characteristics**	
Age	12.0 (0.7)
Sex	
Female	48.8%
Male	51.2%
Race/ethnicity	
White	53.4%
Latino / Hispanic	19.6%
Black	16.5%
Asian	5.6%
Native American	3.2%
Other	1.4%
Nativity	
Youth born in U.S.	96.3%
Youth born outside U.S.	3.7%
Highest parental education	
High school education or less	15.6%
College education or more	84.4%
Household income	
$24,999 or less	16.9%
$25,000 to $49,999	20.2%
$50,000 to $74,999	18.2%
$75,000 to $99,999	16.0%
$100,000 to $199,999	21.7%
$200,000 and greater	7.0%
**Type of discrimination reported**	
Discrimination because of race, ethnicity, or color	4.7%
Been treated unfairly or negatively because of your ethnic background by:	
***Teachers***	
Almost never	92.0%
Rarely	4.3%
Sometimes	2.1%
Often	0.9%
Very Often	0.7%
***Adults outside school***	
Almost never	90.2%
Rarely	6.5%
Sometimes	2.2%
Often	0.6%
Very Often	0.5%
***Students***	
Almost never	74.9%
Rarely	13.1%
Sometimes	7.8%
Often	2.2%
Very Often	2.0%
**Binge eating**	
Binge-eating behaviors	7.9%
Binge-eating disorder diagnosis	1.1%

ABCD Study sociodemographic variables were standardized to match the distribution American Community Survey from the U.S. Census.

**Table 2:** Associations between discrimination and binge-eating behaviors and diagnosis (N=11,075)

**Panel A: Bivariate Model**		
	**Binge-eating behaviors**	**Binge-eating disorder diagnosis**
	OR	OR
	(95% CI)	(95% CI)
Discrimination because of race, ethnicity, or color	2.22***	4.31***
	(1.62 – 3.05)	(2.40 – 7.74)
Been treated unfairly or negatively because of your ethnic background by:		
Teachers	1.20**	1.44**
	(1.06 – 1.36)	(1.16 – 1.79)
Adults outside school	1.19*	1.55**
	(1.03 – 1.37)	(1.19 – 2.01)
Students	1 18***	1 54***
	(1.08 – 1.28)	(1.28 – 1.86)
**Panel B: With Confounding Variables**		
	OR	OR
	(95% CI)	(95% CI)
Discrimination because of race, ethnicity, or color	2 12***	3.31*
	(1.50 – 3.00)	(1.66 – 6.63)
Been treated unfairly or negatively because of your ethnic background by:		
Teachers	1.12	1.26
(0.97–1.23)	(0.98 – 1.60)	
Adults outside school	1.11	1.42*
(0.95 – 1.30)	(1.06 – 1.90)	
Students	1.12*	1.36**
	(1.02 – 1.23)	(1.10 – 1.68)

* indicates p<0.05

** indicates p<0.01

*** indicates significant at <0.001.

ABCD Study sociodemographic variables were standardized to match the distribution American Community Survey from the U.S. Census. Panel B models include sex, race/ethnicity, household income, parent education, and site.

## Data Availability

Data used in the preparation of this article were obtained from the ABCD Study (https://abcdstudy.org), held in the NIMH Data Archive (NDA). Investigators can apply for data access through the NDA (https://nda.nih.gov/).

## Abbreviations

ACEs: Adverse Childhood Experiences
ABCD: Adolescent Brain Cognitive Development study
BED: Binge-eating disorder
KSADS-5: Kiddie Schedule for Affective Disorders and Schizophrenia
BN: Bulimia Nervosa
SD: Standard Deviation
